# Biomarkers in two different animal models of pulmonary arterial hypertension (PAH) under treatment with a cGMP enhancing substance

**DOI:** 10.1186/1471-2210-11-S1-P31

**Published:** 2011-08-01

**Authors:** Julia Grupe, Andrea Neumann, Johannes-Peter Stasch, Kirsten Leineweber

**Affiliations:** 1 Pharma Research Centre, Bayer HealthCare, Wuppertal, Germany; 2 Martin-Luther-University, School of Pharmacy, Halle, Germany

## Background

There is still a need of further biomarkers for an early and correct diagnosis of PAH as mean time between onset of symptoms and diagnosis is two years. We investigated markers for pressure overload and markers for remodeling in two animal models of PAH, in order to differentiate between biomarker regulation due to afterload increase based on remodeling of pulmonary vessels (monocrotaline (MCT)-model) and isolated right ventricular pressure overload (pulmonary artery (PA) banding-model). The cGMP enhancing substance Sildenafil was utilized to investigate a response-to-treatment of the markers.

## Methods

An equivalent study design was used for the MCT- and PA banding-study. Male Sprague Dawley rats (250 g body weight) underwent PA banding surgery or received 60 mg/kg MCT s.c. and were randomized in control, placebo and treatment group (n=12 per group) in each study. Treatment with 100 mg/kg Sildenafil in the drinking water was started on day 14. On day 28 final hemodynamic measurements were performed and organ weights were determined. Blood and right ventricular tissue were withdrawn for detection of ANP, BNP, MMP-2, TIMP-1 and OPN gene expression levels and plasma concentrations.

## Results

An almost equivalent increase of right ventricular systolic pressure (RVP) and right ventricular hypertrophy was determined in both models. Gene expression and plasma concentration of the pressure sensitive markers ANP (atrial natriuretic peptide) and BNP (brain natriuretic peptide) were also increased in both models. Gene expression levels of the remodeling markers TIMP-1 (tissue inhibitor of metalloproteinase-1) and OPN (osteopontin) and to a less extent of MMP-2 (matrix metalloproteinase-2) were elevated in both models, though more pronounced in the MCT-model. Elevated plasma concentrations of MMP-2, TIMP-1 and OPN were determined in the MCT-model, but not in the PA banding-model. A significant response-to-treatment with Sildenafil, which was only observed for the MCT-model, was detected for all evaluated markers.

## Conclusion

Pathophysiological right ventricular pressure overload was well represented by ANP and BNP in plasma, which is a proof of the clinical relevance of those markers. Whereas plasma MMP-2, TIMP-1 and OPN were not sufficient to monitor remodeling processes in the right ventricle due to isolated pressure overload and therefore failed as cardiac specific markers for right ventricular remodeling.

